# The development of a highly sensitive and quantitative SARS-CoV-2 rapid antigen test applying newly developed monoclonal antibodies to an automated chemiluminescent flow-through membrane immunoassay device

**DOI:** 10.1186/s12865-023-00567-y

**Published:** 2023-09-26

**Authors:** Kengo Nishimura, Hiroaki Kitazawa, Takashi Kawahata, Kosuke Yuhara, Takahiro Masuya, Toshihiro Kuroita, Kentarou Waki, Seiichi Koike, Masaharu Isobe, Nobuyuki Kurosawa

**Affiliations:** 1 Bio-Science & Medical Research Unit, Corporate Research Center, TOYOBO CO., LTD., 2-1-1 Katata, Otsu-Shi, Shiga, 520-0243 Japan; 2 Biotechnology Research Laboratory, TOYOBO CO., LTD., 10-24, Toyo-Cho, Tsuruga-Shi, Fukui, 914-8550 Japan; 3 Biotechnology Operating Department, TOYOBO CO., LTD., 1-13-1 Umeda, Kita-Ku, Osaka, 530-0001 Japan; 4 Laboratory of Molecular and Cellular Biology, Graduate School of Science and Engineering for Education, University of Toyama, Toyama-Shi, Gofuku, Toyama, 930-8555 Japan; 5 Laboratory of Molecular and Cellular Biology, Graduate School of Innovative Life Science, University of Toyama, 3190 Gofuku, Toyama-Shi, Toyama, 930-8555 Japan

**Keywords:** SARS-CoV-2, COVID-19, Omicron, Rapid antigen test, Point-of-care test, Monoclonal antibody, Lateral flow immunochromatography

## Abstract

**Background:**

Rapid and accurate diagnosis of individuals with SARS-CoV-2 infection is an effective way to prevent and control the spread of COVID-19. Although the detection of SARS‐CoV‐2 viral RNA by RT‐qPCR is the gold standard for COVID-19 testing, the use of antigen-detecting rapid diagnostic tests (Ag-RDTs) is emerging as a complementary surveillance tool as Omicron case numbers skyrocket worldwide. However, the results from Ag-RDTs are less accurate in individuals with low viral loads.

**Results:**

To develop a highly sensitive and accurate Ag-RDT, 90 monoclonal antibodies were raised from guinea pigs immunized with SARS CoV-2 nucleocapsid protein (CoV-2-NP). By applying a capture antibody recognizing the structural epitope of the N-terminal domain of CoV-2-NP and a detection antibody recognizing the C-terminal tail of CoV-2-NP to an automated chemiluminescence flow-through membrane immunoassay device, we developed a novel Ag-RDT, CoV-2-POCube. The CoV-2-POCube exclusively recognizes CoV-2-NP variants but not the nucleocapsid proteins of other human coronaviruses. The CoV-2-POCube achieved a limit of detection sensitivity of 0.20 ~ 0.66 pg/mL of CoV-2-NPs, demonstrating more than 100 times greater sensitivity than commercially available SARS-CoV-2 Ag-RDTs.

**Conclusions:**

CoV-2-POCube has high analytical sensitivity and can detect SARS-CoV-2 variants in 15 min without observing the high-dose hook effect, thus meeting the need for early SARS-CoV-2 diagnosis with lower viral load. CoV-2-POCube is a promising alternative to currently available diagnostic devices for faster clinical decision making in individuals with suspected COVID-19 in resource-limited settings.

**Supplementary Information:**

The online version contains supplementary material available at 10.1186/s12865-023-00567-y.

## Background

COVID-19 is a disease caused by infection with severe acute respiratory syndrome coronavirus 2 (SARS-CoV-2), which causes mild upper respiratory symptoms in most cases but can lead to the development of bilateral pneumonia with acute respiratory distress in some [1]. COVID-19 and other respiratory infections, such as influenza, have many common signs and symptoms, making it difficult for clinicians to identify the pathogen based on symptoms alone. Quantitative reverse transcription polymerase chain reaction (RT-qPCR) tests that amplify SARS-CoV-2 viral RNA are the gold standard for COVID-19 diagnosis. Although RT-qPCR is highly sensitive and specific, it has several limitations, including a long turnaround time and labor-intensive protocol, and requires specialized equipment [2]. In addition, the test results do not necessarily indicate the presence of viable virus because RT-qPCR only detects viral RNA. This can lead to unnecessary isolation of noninfectious or disease-recovered individuals. As the number of Omicron case skyrocket around the world, RT-qPCR will not be able to meet the high demand for testing to control the pandemic situation. These facts point to the need for rapid and sensitive diagnostic tests that can be used in the field when suspected COVID-19 cases are identified [3]. Antigen-detecting rapid diagnostic tests (Ag-RDTs), which use monoclonal antibodies (mAbs) to detect the presence of the SARS-CoV-2 viral protein, are widely used as point-of-care tests with results available within 30 min.

SARS-CoV-2 contains four major structural proteins: the spike, envelope, membrane and nucleocapsid protein (NP). SARS-CoV-2 nucleocapsid protein (CoV-2-NP) is a 46 kDa phosphoprotein consisting of the following: an N-terminal domain, an RNA binding domain, a central linker, a dimerization domain and a C-terminal tail [4]. The abundance and low mutation rate of CoV-2-NP make it an ideal antigen for the detection of SARS-CoV-2 by Ag-RDTs [5, 6]. Most SARS-CoV-2 Ag-RDTs use sandwich lateral flow immunochromatography (LFIC) to detect CoV-2-NP to primarily determine the results visually. These tests can be used during a specific timeframe after symptom onset when the virus is present in the respiratory tract at some level. However, many of these tests are generally inappropriate for individuals with a low viral load due to their low sensitivity [7–10]. To improve the limitations of Ag-RDTs, several methods have been developed, including electrochemical biosensors, surface plasmon resonance and microfluidic strips [11–13]. However, many of them have not yet been put into practical use due to the complexity of the technology. Therefore, a rapid and sensitive method for COVID-19 diagnosis is urgently needed, especially in resource-poor and remote settings.

One of the key solutions to improve the performance of Ag-RDTs is the development of mAbs that can strongly and specifically react to SARS-CoV-2 antigens. We have recently shown that guinea pigs can be a novel source of monoclonal antibodies. Because guinea pigs have a completely different B-cell *repertoire than mice, this animal* may offer novel solutions for the production of high-affinity antibodies against CoV-2-NP that cannot be produced in mice [14, 15]. In this study, we developed mAbs from guinea pigs immunized with CoV-2-NP and selected highly specific epitope-characterized mAbs suitable for the detection of CoV-2-NP. By applying the mAbs to our previously developed fully automated chemiluminescence flow-through membrane immunoassay device, POCube®, we successfully developed a highly sensitive, specific, and quantitative SARS-CoV-2 antigen test, CoV-2-POCube [16]. CoV-2-POCube exclusively detects a panel of CoV-2-NP variants, including Wuhan Hu-1, Alpha (B.1.351), Beta (B.1.351), Gamma (P.1.2), Delta (B.1.617.2) and Omicron (B.1.1.529 and BA.2), but not the NP of SARS-CoV and other human coronaviruses. The performance of CoV-2-POCube is more than 100 times greater than that of commercial SARS-CoV-2 Ag-RDTs, with a limit of detection sensitivity (LOD) of 0.20 ~ 0.66 pg/mL [10, 17]. Because CoV-2-POCube is compact and requires only a few operation steps, it is suitable for faster clinical decision-making in individuals suspected of COVID-19 infection in resource-limited settings.

## Results

### Development and screening of mAbs against CoV-2-NP

**Fig. 1:**
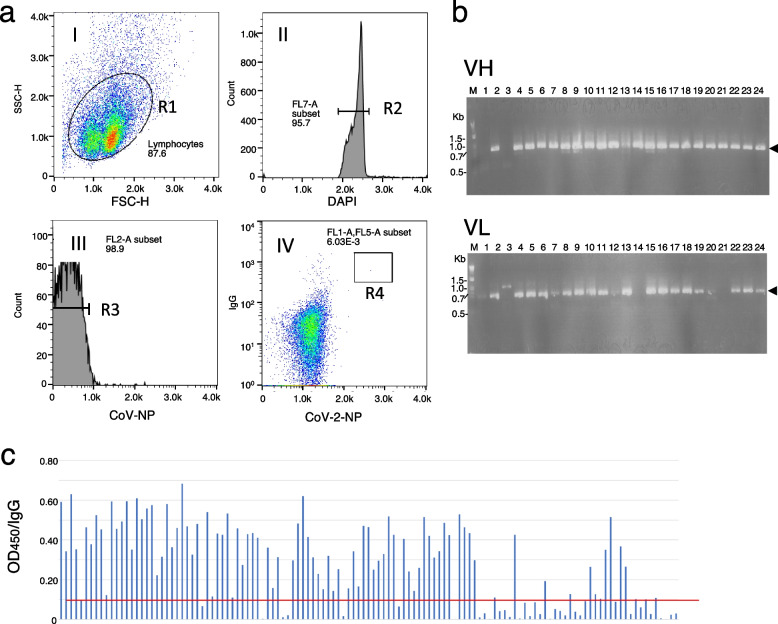
Single-cell-based development of anti-CoV-2-NP mAbs. **a** FACS gating strategy for the isolation of Wuhan CoV-2-NP-specific plasma cells. Plots (I)–(IV) represent the sequential gating strategy. (I) FSC vs. SSC with gate R1 represents lymphocytes. (II) Single cells were selected via DAPI staining (R2). (III) Cells labeled with CoV-NP were excluded from the R3 gate. (IV) The CoV-2-NP^High^ CoV-NP^Low^ and anti-guinea pig IgG^High^ fraction was defined as Wuhan CoV-2-NP-specific plasma cells (R4 gate). Percentages show the content of each cellular subpopulation among the total number of lymph node cells. **b** Representative agarose gel electrophoresis of the cognate pairs of immunoglobulin heavy chain variable (VH) and light chain kappa variable (VL) genes amplified from the single-cell-sorted R4-gated cells in (A). Line M: 1 kb DNA ladder (New England Biolab). Arrowheads indicate the size of the amplified DNA fragments. **c** The antigen specificity of the mAbs produced from the R4-gated cells. Cognate pairs of immunoglobulin heavy and light chain genes were cotransfected into HEK293 cells, and the CoV-2-NP binding activities of the expressed mAbs (5 ng) were analyzed by ELISA. The red line indicates the cutoff line for the screening

To develop a sensitive and quantitative SARS-CoV-2 Ag-RDT, we used Wuhan-Hu-1 (Wuhan) CoV-2-NP as an immunogen for mAb production. After immunization of guinea pigs with Wuhan CoV-2-NP, isolated lymph node cells were intracellularly stained with DyLight 488-conjugated Wuhan CoV-2-NP, anti-IgG and DAPI. The cells were further stained with DyLight 550-conjugated SARS-CoV NP (CoV-NP) to exclude plasma cells cross-reacting with CoV-NP. A fraction of the Wuhan CoV-2-NP-specific plasma cells, characterized as Wuhan CoV-2-NP^High^, CoV-NP^Low^ and anti-IgG^High^, was single-sorted by fluorescence-activated cell sorting (FACS) (Fig. 1a).

**Fig. 2:**
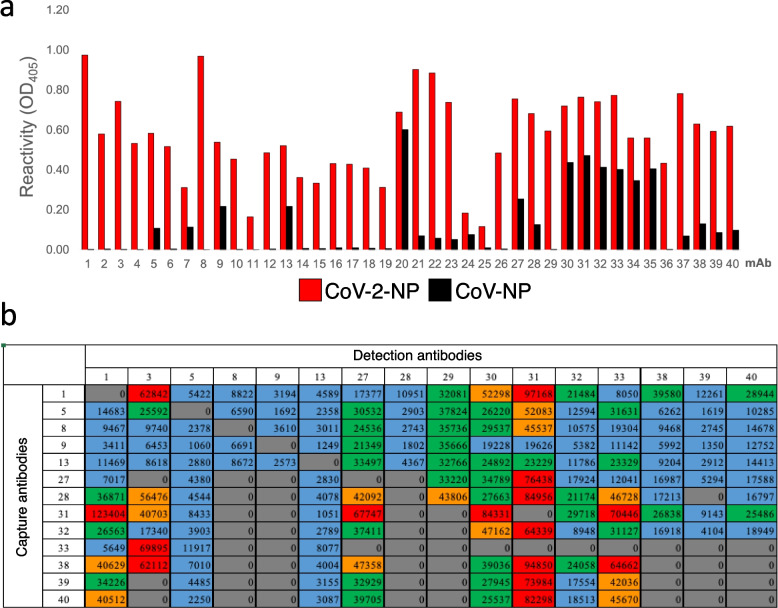
The selection of appropriate detection and capture antibodies using a sandwich assay. **a** Reactivity and specificity of the candidate mAbs. Forty candidate mAbs were tested with either Wuhan CoV-2-NP or CoV-NP to evaluate cross-reactivity by ELISA. Data are expressed as the mean of two measurements. **b** Pairwise analysis of mAbs against Wuhan CoV-2-NP. Sandwich assay was used for pairwise coupling of thirteen capture mAbs against sixteen detection mAbs. The numbers inside the grid are the luminescence activities. The heatmap represents the performance of antibody pair. Red, > 80,000; orange, 80,000–60000; green, 59,999–40,000; blue, 39,999–20,000; gray, not determined. Data are expressed as the mean of two measurements. The highest luminescence was considered as the optimal mAb set

Single-cell-based rapid amplification of 5’ cDNA ends PCR resulted in the amplification of cognate pairs of immunoglobulin heavy chain variable and kappa light chain variable genes (Fig. 1b). DNA transfection of the cognate pairs of full-length immunoglobulin heavy and light chain genes into HEK293 cells resulted in the production of mAbs. These mAbs were evaluated for reactivity with Wuhan CoV-2-NP by enzyme-linked immunosorbent assay (ELISA) and cell ELISA, which showed that a total of 90 mAbs bound to the antigen (Fig. 1c and Additional file 1: Fig. S1). Forty candidate mAbs were then selected for further study based on their unique complementarity-determining region 3 sequences. The cross-reactivities of these mAbs with CoV-NP were further analyzed by ELISA, which revealed that ~ 50% of the mAbs were specific for CoV-2-NP (Fig. 2a and Additional file 2: Table S1).

### Selection of mAbs for use in the antigen capture flow-through membrane immunoassay

To select the optimal mAb pair for the specific detection of Wuhan CoV-2-NP, representative mAbs were selected from the forty candidates based on their reactivity, and pairwise analysis was performed by sandwich assay. As shown in Fig. 2b, the analysis revealed a variety of signal intensities among the mAb combinations. For example, when #1, #3, #27, #30, #31 or #33 was used as the detection mAb, a high signal was obtained in combination with various capture mAbs. Finally, the pairing of #1 and #31 gave the highest sensitivity. Since #1 is specific for Wuhan CoV-2-NP and #31 reacts with Wuhan CoV-2-NP and CoV-NP, we selected #1 as the detection mAb and #31 as the capture mAb for subsequent experiments.

**Fig. 3:**
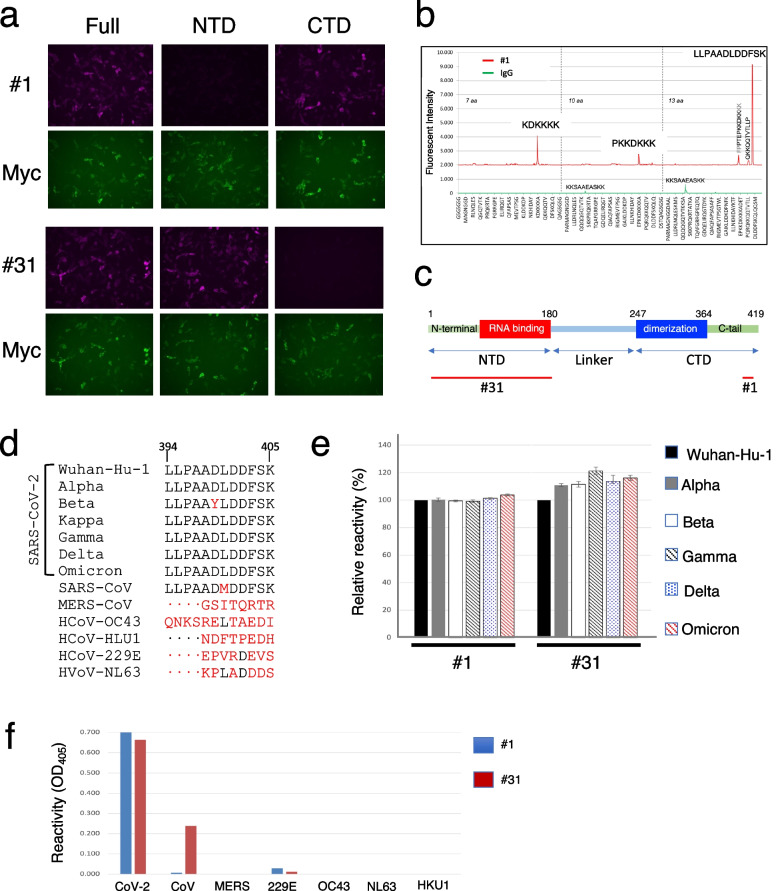
Epitope mapping of mAbs. **a** Antibody epitope analysis of Wuhan CoV-2-NP by cell ELISA. HEK293 cells expressing either the full-length (Full), N-terminal domain (NTD) or C-terminal domain (CTD) of myc-tagged Wuhan CoV-2-NP were stained with the indicated mAbs. Representative florescence images are shown. **b** Epitope mapping of #1 using solid-phase peptide arrays. Solid-phase supports containing synthetic peptides spanning the C-terminus of Wuhan CoV-2-NP were screened for reactivity with #1. Major epitopes were defined as having greater than ten times the reactivity of guinea pig IgG. **c** Schematic diagram of the CoV-2-NP domain. Each mAb epitope determined by peptide array and/or immunostaining is shown with a red bar. **d** Epitope residues of #1 on CoV-2-NPs of SARC-CoV-2 variants and NPs of other human coronaviruses. Epitope residues that are conserved are shown in black, and those that are not conserved are shown in red. **e** Cross-reactivity of mAbs with CoV-2-NPs of SARS-CoV-2 variants. The indicated mAb was reacted with a series of CoV-2-NPs immobilized on plates, and the binding was analyzed by ELISA. The results are expressed as the mean ± SD of four replicates relative to Wuhan CoV-2-NP. **f** Cross reactivity of #1 and #31 with NPs of human coronaviruses. The indicated mAb was reacted with a series of NPs immobilized on plates, and the binding was analyzed by ELISA. The results are expressed as the mean of two replicates

Next, we tried to determine the epitope of these mAbs by using HEK293 cells expressing either the N-terminal domain (aa 1–180) or the C-terminal domain (aa 247–419) of Wuhan CoV-2-NP. It was found that #1 reacted with the C-terminal domain and #31 reacted with the N-terminal domain (Fig. 3a).

For further characterization of the mAbs, linear epitope mapping was performed by using a peptide microarray. It was shown that #1 recognized the C-terminal tail of Wuhan CoV-2 NP (LLPAADLDDFSK) but not to CoV-NP (LLPAADMDDFSK), suggesting that the mAb recognizes the lysine at position 400. #31 did not bind any peptides, supporting its conformational epitope (Fig. 3b). Because these mAbs were developed against Wuhan CoV-2-NP as an antigen, this raises the possibility that the antigen recognition capacity may be impaired due to mutations in SARS-CoV-2 variants. ELISA analysis of these mAbs against CoV-2-NPs of seven SARS-CoV-2 variants showed that the binding capacity of #1 was not lost for all SARS-CoV-2 variants examined, although Beta variant has a D399Y mutation in CoV-2-NP, suggesting that the minimum epitope of #1 may be LDDFSK. #31 reacted with all CoV-2-NPs of SARS-CoV-2 variants (Fig. 3c, d and e). Based on their cross-reactivity profiles, the #1/#31 set was expected to have the potential to detect a variety of SARS-CoV-2 variants. To further ensure the specificity of the two mAbs, the cross-reactivity against NPs of other human coronaviruses, including MERS, 229E, OC43, NL63 and HKU1, was examined. As shown in Fig. 3f, none of the mAbs reacted with the NPs of the other coronaviruses, except for the marginal level of binding to 229E-NP.

**Fig. 4:**
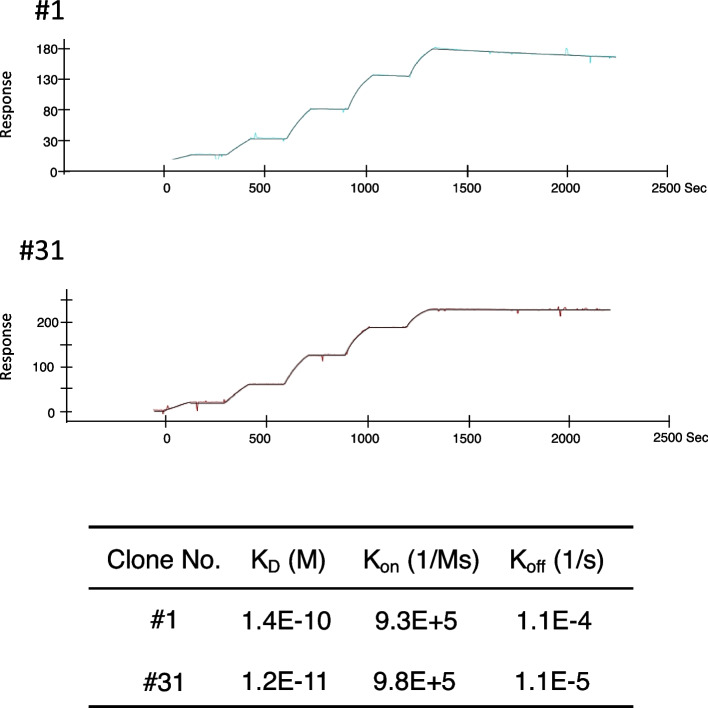
Antibody affinity determination by SPR

Surface plasmon resonance (SPR) was used to detail the kinetic properties and affinities of the selected mAbs. The kinetic analyses highlight the fast association (k_on_), and slow dissociation (k_off_) rate for the interaction with Wuhan CoV-2-NP, resulting in equilibrium dissociation constants (K_D_ values) of 1.4 × 10^–10^ M for #1 and 1.2 × 10^–11^ M for #31 (Fig. 4).

Biotinylated Wuhan CoV-2-NP was immobilized on streptavidin sensor chips, and antibodies at different dilutions (0 ~ 27 nM) were allowed to bind. Representative SPR sensorgrams are shown. The results are expressed as the mean of three replicates.

### Development of a highly sensitive SARS-CoV-2 Ag-RDT, CoV-2-POCube

**Fig. 5:**
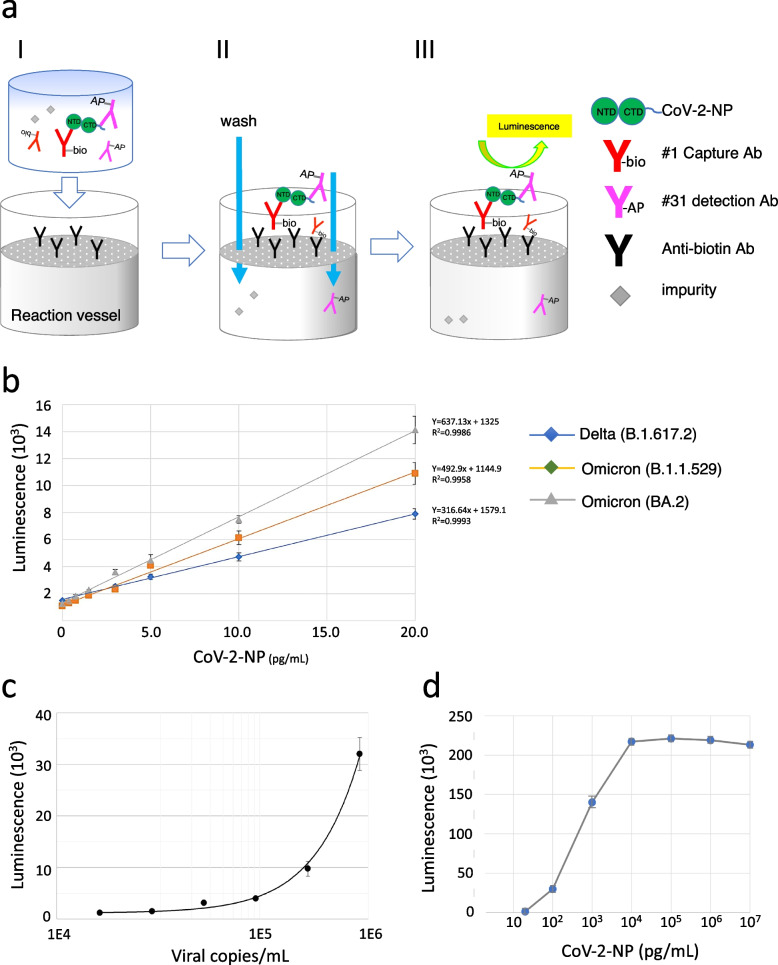
Determination of the technical sensitivity of CoV-2-POCube. **a** A schematic of CoV-2-POCube. Immunocomplex formation in the test sample followed by the sample loaded into a reaction vessel (I). Immunocomplex trapping on an anti-biotin antibody-coated membrane followed by washing to remove contaminants (II). Luminescence signal measurement (III). Reaction time requires 10 min for (I) and 5 min for (II) ~ (III), totaling 15 min. **b** The calibration curve of CoV-2-POCube for CoV-2-NP detection. Serially diluted recombinant CoV-2-NP of Delta or Omicron variant were subjected to CoV-2-POCube analysis. **c** COV-2-POCube signal intensity of serially diluted heat-inactivated Wuhan SARS-CoV-2 particles. Each dilution was analyzed for CoV-2-NP using a COV-2-POCube. **d** Correlation between COV-2-POCube signal and CoV-2-NP concentrations. COV-2-POCube signal intensity of serially dilute Delta CoV-2-NPs are shown. All data are presented as the mean ± SD of three replicates

POCube® is a fully automated, compact chemiluminescence flow-through membrane immunoassay device developed by Toyobo Co. Ltd, and this device is intended to support the point-of-care testing system in clinics [16, 18]. A schematic giagram of the device is shown in Fig. 5a. In this device, the capture and detection mAbs bind to CoV-2-NP to form an immunocomplex.

As the mixture passes through the membrane, the immunocomplex binds to a filter immobilized with a polyclonal goat anti-biotin antibody. After washing the filter to remove unconjugated mAbs and sample contaminants, the addition of an alkaline phosphatase chemiluminescent substrate generates a luminescent signal on the filter. We applied biotin-conjugated #31 as a capture antibody and alkaline phosphatase-conjugated #1 as a detection antibody to POCube® and analyzed the sensitivity and quantitation of this assay by using serially diluted recombinant Delta CoV-2-NP. As shown in Fig. 5b, the quantitative linear range was 1.5–20.0 pg/mL with a reliable correlation coefficient (R2 = 0.999), while the LOD value was 0.66 pg/mL. Nearly the same results were obtained when Omicron B.1.1.529 and BA.2 were tested, with LODs of 0.37 pg/mL and 0.20 pg/mL, respectively. A subsequent study using heat-inactivated SARS-CoV-2 Delta strain particles showed that the LOD of the assay was 8.0 × 10^4^ copies/mL (Fig. 5c). To determine whether the CoV-2-POCube suffered from a high dose hook effect, increasing concentrations of CoV-2-NP were tested up to a concentration of 10 μg/mL. As shown in Fig. 5d, CoV-2-POCube signal increased with increasing CoV-2-NP concentration until it reached the plateau range (> 10 ng/ml). No clear high dose hook effect was observed up to 10 μg/mL, which can be converted to a viral copy number of 6.8 × 10^9^ copies/mL [8].

## Discussion

LFIC Ag-RDTs have been widely used for diagnosis due to their ease of use, rapid results and suitability for point-of-care testing. However, many tests are qualitative, with positive or negative results judged by the naked eye, which lacks objectivity and can lead to misdiagnosis. To date, various efforts have been made to improve the sensitivity and quantitation of Ag-RDTs by using either reader systems and/or additional reagents [11, 19–23]. However, the most important factor in overcoming the limitation associated with LFIC Ag-RDTs is the development of a quantitative sandwich immunodetection format, which is highly dependent on the sensitivity and overall specificity of a pair of mAbs.

It was found that #1 binds to all types of CoV-2-NP variants tested, but not to NPs of other human coronaviruses, as it recognizes the C-terminal tail, which is not conserved among other human coronaviruses. These characteristics suggest that this mAb is suitable for use as a detection antibody. The capture antibody selected, #31, recognizes the structural epitope in the N-terminal domain of CoV-2-NP and CoV-NP, but it does not compromise the specific detection of CoV-2-NP when used in combination with #1. Each mAb binds to a spatially distant epitope, increasing the sensitivity and specificity of the assay. In addition, these mAbs are ideal for use in an Ag-RDT due to their high k_on_ and low k_off_, characteristics that allow them to bind rapidly to the antigen and maintain strong binding, enabling rapid and sensitive detection of CoV-2-NP. In addition, we applied the mAb set to a chemiluminescent flow-through membrane immunoassay format, which can wash out contaninants in the sample to reduce background noise, thereby avoiding false positive signals and maximizing the detection signal. Although #1 and #31 showed weak cross-reactivity to 229E-NP, as shown in Fig. 3f, the intensity was only 1/500–1/250 times of CoV-2, so it is not expected to have a significant effect on CoV-2-POCube testing using this antibody set. The LOD of CoV-2-POCube was more than 100 times higher than those of commercially available Ag-RDTs (Table 1) [10, 17, 23, 24]. Therefore, it may be possible to detect the virus during the early phase of illness, which is critical for the early diagnosis of COVID-19.

**Table 1:** Sensitivity of Ag-RDTs for COV-2-NP Detection

Product name	Entity	LOD for CoV2-NP	Mechanism	References
Espline SARS-CoV-2 rapid antigen test	Fujirevio	25 pg/mL	Enzyme linked LFIC	[17]
COVID-19 (SARS-CoV-2) Antigen Test Kit	Deepblue	5.5 μg/mL	LFIC	[24]
COVID-19 (SARS-CoV-2) Antigen Test Kit	EazyDiagnosis	550 pg/mL	LFIC	[24]
COVID-19 TO-GO	Exotest	1 μg/mL	LFIC	[24]
ImmunoArrow SARS-CoV-2 antigen test kit	Toyobo	25 pg/mL	LFIC	[17]
COVID-19 and Influenza A + B Antigen Combo Rapid Test	Flowflex	100 pg/mL	LFIC	[17]
Fuji Dri-chem immuno AG cartridge COVID-19 Ag	FUJIFILM Corporation	10 pg/mL	Silver amplification LFIC	[17]
Elecsys® SARS-CoV-2 antigen assay	Roche Diagnostics	10 pg/mL	Florescent nanoparticles LFIC	[23]
SD Biosensor	Roche Diagnostics	5 ng/mL	LFIC	[22]
Panbio COVID-19 Ag Rapid Test	Abbott	5 ng/mL	LFIC	[10]
COVID-19 Ag Respi-Strip	Coris BioConcept	25 ng/mL	LFIC	[10]
CoV-2-POCube		0.02 ~ 0.66 pg/mL	Chemiluminescent immunoassay	This work

The limitation of this study is that we did not test CoV-2-POCube with clinical samples because our main objective was the development of mAbs and application in POCube® to evaluate the sensitivity and quantitation for the detection of CoV-2-NP. Further research is needed before it can be considered for clinical application.

## Conclusions

A highly sensitive Ag-RDT, CoV-2-POCube, applying the newly developed mAb set onto an automatic immunoassay device was developed. The key feature of CoV-2-POCube is that the platform uses a chemiluminescent flow-through membrane immunoassay format that washes out contaminants in the sample and reduces background noise, thereby maximizing signal-to-noise ratio. These features maximize the benefits of the epitope-characterized high affinity antibody set that exclusively recognizes CoV-2-NPs of SARS-CoV-2 variants but not the nucleocapsid proteins of SARS-CoV and other human coronaviruses. The capabilities of CoV-2-POCube include its high speed of detection (15 min), higher analytical sensitivity (LOD = 0.20 ~ 0.66 pg/mL) without observing the hook effect up to 10 μg/mL. Although this technology lags in the ability to detect multiple samples at once, our experimental results demonstrate the effectiveness of a highly sensitive and rapid point-of-care antigen-based test for SARS-CoV-2, with better performance compared to currently available diagnostic devices. CoV-2-POCube is suitable for rapid clinical decision-making in people with suspected COVID-19 in limited-resource settings.

## Materials and methods

Wuhan CoV-2-NP was obtained from RayBiotec (#230–30164). Avi-His-Tag-Alpha (B.1.1.7 Variant, #100,986), Beta (B.1.351 Variant, #100,985), and Gamma (P.1 Variant, #100,987), CoV-2-NP were obtained from BPS Bioscience. Delta (B.1.617.2, D63G, R203M, G215C, D377Y, #40,588-V07E32) and Omicron (B.1.1.529, #NUN-C52Ht) CoV-2-NP were obtained from Sino Biological and Acro Biosystems, respectively. Female Hartley guinea pigs were purchased from Japan SLC, Inc. DyLight 488 and 550 microscale antibody labeling kits, DyLight 650-labeled anti-guineapig IgG and DAPI were obtained from ThermoFisher Scientific. Horseradish peroxidase-labeled anti-guinea pig antibody was obtained from Abcam. Heat-inactivated SARS-CoV-2 particles were acquired from Zeptmetrix (508,147 viral particles/mL). The plasmid encoding myc-tagged Wuhan CoV-2-NP (pCMV-CoV-2-NP) was obtained from Sino Biological.

### Immunization and isolation of antigen-specific plasma cells

Anesthetized guinea pigs were immunized three times intramuscularly at the tail base with 100 μl of a 50:50 water-in-oil TiterMax Gold adjuvant emulsion containing 50 μg of wild-type CoV-2-NP. One week after the final immunization, guinea pigs were euthanized with intraperitoneal injection of 100 mg/kg sodium pentobarbital solution. Iliac lymph nodes were surgically removed from the euthanized animals and used for the isolation of antigen-specific plasma cells as previously described [25]. Briefly, lymph node cells were fixed with ice-cold phosphate-buffered saline (PBS) containing 2% paraformaldehyde, incubated for 10 min on ice, suspended in PBS containing 0.1% Triton X-100 (PBST) and stained with DyLight 488-labeled CoV-2-NP, DyLight 550-labeled CoV-NP, DyLight 650-labeled anti-guinea pig IgG and DAPI. CoV-2-NP-specific plasma cells, defined as COV-2-NP^High^, COV-NP^Low^ and IgG^High^, were single-sorted using a JSAN Cell Sorter (Bay Bioscience). Flow cytometry data were analyzed using FlowJo Software version 10.7.1 (BD Biosciences).

### Generation of monoclonal antibodies

Amplification of immunoglobulin heavy and light chain variable genes by rapid amplification of 5’ cDNA ends PCR was performed as previously described [26]. High-throughput production of recombinant antibodies was performed by TS-jPCR as previously described [26]. Briefly, cognate pairs of linear immunoglobulin heavy and light chain genes were cotransfected into HEK293 cells, and the cell culture supernatant was used for enzyme-linked immunosorbent assay (ELISA) and cell ELISA four days after transfection. For large-scale antibody production, immunoglobulin heavy and light chain genes were inserted into pET-IgG and pET-IgK vectors by target-selective homologous recombination cloning as described previously [27]. Transient transfection of the plasmids into CHO-S cells was performed with the CHOgro High Yield Expression System (Takara Bio). Recombinant mAbs were purified from the CHO cell culture medium by using MabCaptureC™ Protein A chromatography resin (ThermoFisher Scientific). The purified antibodies were labeled with either alkaline phosphatase or biotin by using Alkaline Phosphatase Labeling Kit-SH or Biotin Labeling Kit -NH2 according to the manufacturer’s instructions, respectively (Dojindo).

### ELISA

Each well of 96-well plates (Corning) immobilized with 15 ng of COV-2-NP or COV-NP were blocked with PBS containing 1% bovine serum albumin (BSA). Crude or purified antibodies (5 ng) were added to the plates, incubated at 25 °C for 120 min to allow the antibody to react with the antigen, and then washed three times with PBST. Horseradish peroxidase-labeled anti-guinea pig antibody was added to the plates and incubated at 25 °C for 60 min. The plates were washed three times with PBST and developed with TMB (SURMODICS). The reaction was stopped with 1 N sulfuric acid, and the absorbance values at 450 nm and 620 nm were measured. PBS was used as a blank control.

### Cell ELISA

A plasmid encoding the N-terminal domain or C-terminal domain of CoV-2-NP was constructed by PCR using pCMV-CoV-2-NP as a template. Each plasmid was transfected into HEK293 cells using FuGENE 6 Transfection Reagent (Promega) and cultured for two days. After fixation with PBS containing 4% paraformaldehyde and permeabilization with PBST, cells were stained with anti-CoV-2-NP mAb (0.1 µg/mL) and anti-Myc mAb (0.1 µg/mL), and signals were developed with goat anti-guinea pig IgG DyLight 647 and goat anti-mouse IgG DyLight 488. Images were captured with an Operetta High Content Imaging System (PerkinElmer) or a BZ-X700 All-in-one fluorescence microscope (KEYENCE).

### Peptide microarray analysis of the linear epitopes

Epitope mapping of mAbs against CoV-2-NP was performed by PEPperMAP peptide microarray F-PEP-CEM-3 (Filgen). The CoV-2-NP sequence was converted into 7, 10 or 13 amino acid peptides with peptide-peptide overlaps of 6, 9 or 12 amino acids. Microarrays containing 672 different CoV-2-NP peptides were incubated with the purified mAb (10 µg/ml) for 16 h at 4 °C with orbital shaking at 140 rpm, and the signal was developed by goat anti-guinea pig IgG (H + L) DyLight 680. Images were captured with an Innopsys InnoScan 710-IR microarray scanner.

### Surface plasmon resonance (SPR) analysis

All SPR experiments were performed with a Biacor T100 (Cytiva). Biotinylated CoV-2-NP was immobilized on streptavidin SA sensor chips (Cytiva). Briefly, two flow cells were prepared, one of which served as a negative control, while biotinylated CoV-2-NP was injected into the other to obtain an immobilization level of 150 response units (RU) at a flow rate of 30 μl/min. The interaction assays involved injections of 5 different dilutions of mAb (0, 0.3, 1, 3, 9 nM), followed by a 3 min washing step with HBS-EP + buffer at a flow rate of 30 μl/min to induce the dissociation of the complexes formed. At the end of each cycle, the sensor chip surface was regenerated by injection of 0.1 M citric acid, pH 3. The sensorgrams corresponding to the CoV-2-NP signal were subtracted from the negative control. The single cycle kinetic curves were fitted to a bivalent analyte model to estimate the association rate (k_on_), dissociation (k_off_) rate and equilibrium dissociation constants (K_D_).

### Sandwich assay and POCube® setting

Biotin-labeled anti-SARS-CoV-2 capture mAb (50 ng) and alkaline phosphatase-labeled anti-SARS-CoV-2 detection mAb (3.2 ng) were mixed with different dilutions of recombinant CoV-2-NP in 65 µL of sample buffer (100 mM Tris–HCl buffer containing 0.1% BSA, pH 8.5) to form an immune complex at 40℃ for 10 min. The immune complex was captured on a filter on which an anti-biotin goat polyclonal antibody (2.4 μg) was immobilized. The filter was then washed with 160 µL of a washing solution (10 mM MOPS buffer containing 0.05% Tween 20, pH 7.2), and a luminescence signal was produced by adding a chemiluminescent substrate for alkaline phosphatase (APS-5, Lumigen) to the filter. The signal was measured using a photomultiplier tube settled in POCube®. Sample buffer was used as a blank control.

### Statistical analysis

The limit of detection (LOD) was determined as the mean value of the negative controls (n = 10) plus 3.3 times the standard deviation.

### Supplementary Information

**Additional file 1:** **Fig. S1.** Cell-ELISA screening of mAbs raised from immunized guinea pigs.

**Additional file 2: Table S1.** The specific activity of forty candidate mAbs determined by ELISA and immunostaining.

## Data Availability

Datasets used and/or analyzed during the current study are available from the corresponding author on reasonable request.

## Abbreviations

Ag-RDT: Antigen-detecting rapid diagnostic test
CoV-2-NP: SARS-CoV-2 nucleocapsid protein
CoV-NP: SARS-CoV nucleocapsid protein
ELISA: Enzyme-linked immunosorbent assay
FACS: Fluorescence-activated cell sorting
LFIC: Lateral flow immunochromatography
LOD: Limit of detection
mAb: Monoclonal antibody
RT-qPCR: Quantitative reverse transcription polymerase chain reaction
SARS-CoV-2: Severe acute respiratory syndrome coronavirus 2
SPR: Surface plasmon resonance
