# Supplementary material for: The development of a highly sensitive and quantitative SARS-CoV-2 rapid antigen test applying newly developed monoclonal antibodies to an automated chemiluminescent flow-through membrane immunoassay device
Source: BMC Immunol. 2023 Sep 26;24:34. doi: 10.1186/s12865-023-00567-y (PMC10523765; doi:10.1186/s12865-023-00567-y)
Supplement: Supplementary file 1 — Additional file 1: Fig. S1. Cell-ELISA screening of mAbs raised from immunized guinea pigs. [file 12865_2023_567_MOESM1_ESM.pdf]

Additional file 1: Fig. S1. Cell-ELISA screening of mAbs raised from immunized guinea pigs

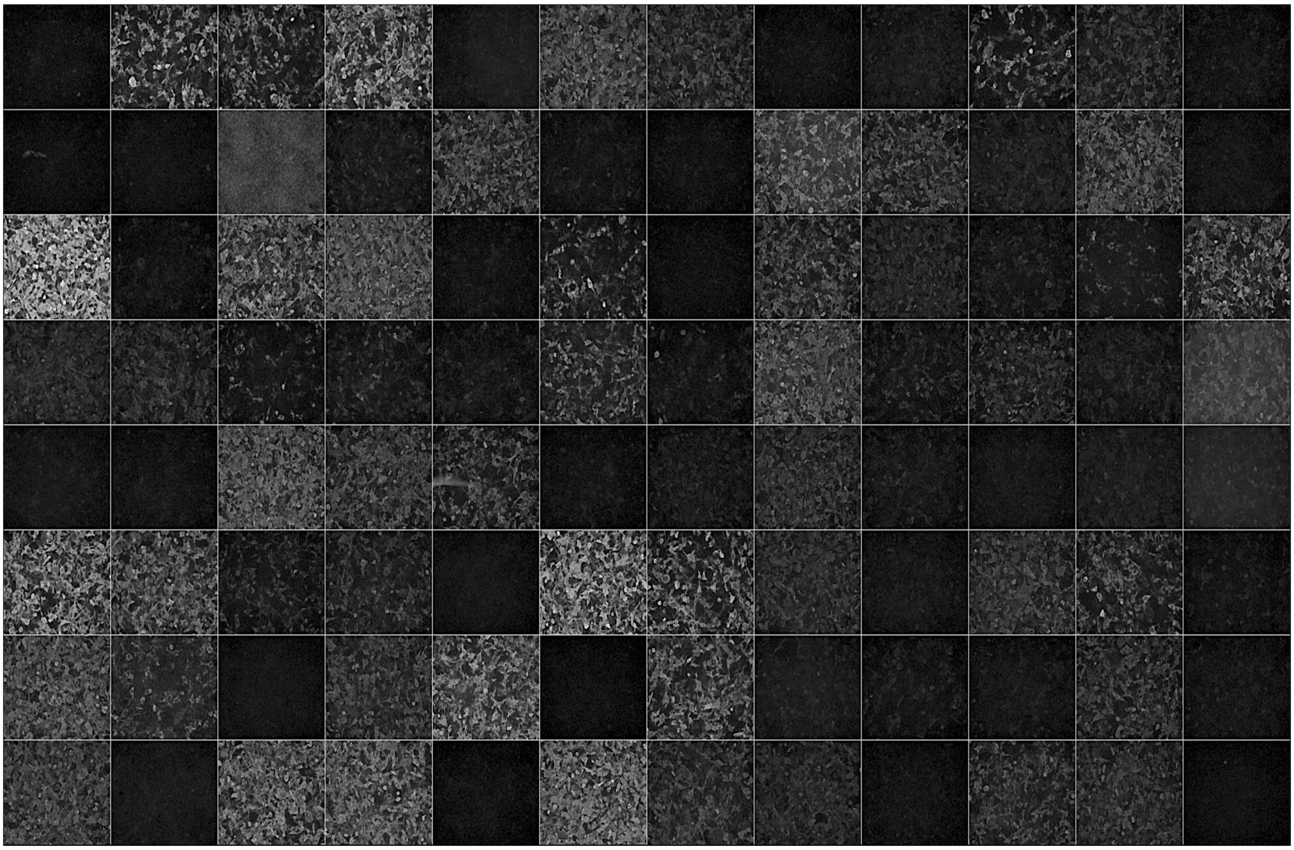

Cell-ELISA screening of mAbs raised from immunized guinea pigs. Each HEK293 cell culture medium containing a mAb was used to assess its reactivity with CoV-2-NP expressed in HEK293 cells. Representative immunofluorescent images taken by the Operetta High Content Imaging System are shown.
