# Supplementary material for: The development of a highly sensitive and quantitative SARS-CoV-2 rapid antigen test applying newly developed monoclonal antibodies to an automated chemiluminescent flow-through membrane immunoassay device
Source: BMC Immunol. 2023 Sep 26;24:34. doi: 10.1186/s12865-023-00567-y (PMC10523765; doi:10.1186/s12865-023-00567-y)
Supplement: Supplementary file 2 — Additional file 2: Table S1. The specific activity of forty candidate mAbs determined by ELISA and immunostaining. [file 12865_2023_567_MOESM2_ESM.docx]

Table S1. The specific activity of forty candidate mAbs determined by ELISA and immunostaining.

| Clone  NO. | Reactivity (OD_405_) | | Specificity; Ratio of SARS-CoV-2/SARS-CoV | Epitope |
| --- | --- | --- | --- | --- |
|  | SARS-CoV-2 | SARS-CoV |  |  |
| 1 | 0.976 | 0.002 | 406.7 | 390-405 |
| 2 | 0.581 | 0.004 | 145.3 | 247-419 |
| 3 | 0.743 | 0.003 | 247.7 | 250-257 |
| 4 | 0.533 | 0.002 | 266.5 | ND |
| 5 | 0.584 | 0.108 | 5.4 | ND |
| 6 | 0.518 | 0.004 | 129.5 | 247-419 |
| 7 | 0.312 | 0.114 | 2.7 | ND |
| 8 | 0.970 | 0.001 | 970.0 | 1-180 |
| 9 | 0.539 | 0.217 | 2.5 | 1-180 |
| 10 | 0.455 | 0.003 | 151.7 | 1-180 |
| 11 | 0.166 | 0.001 | 166.0 | ND |
| 12 | 0.486 | 0.004 | 121.5 | 1-180 |
| 13 | 0.522 | 0.217 | 2.4 | 1-180 |
| 14 | 0.363 | 0.007 | 51.9 | ND |
| 15 | 0.334 | 0.006 | 55.6 | ND |
| 16 | 0.432 | 0.010 | 43.2 | ND |
| 17 | 0.429 | 0.010 | 42.9 | ND |
| 18 | 0.410 | 0.008 | 51.3 | ND |
| 19 | 0.313 | 0.006 | 52.2 | ND |
| 20 | 0.689 | 0.602 | 1.1 | 1-180 |
| 21 | 0.903 | 0.070 | 13.0 | ND |
| 22 | 0.886 | 0.058 | 15.2 | ND |
| 23 | 0.738 | 0.052 | 14.2 | ND |
| 24 | 0.185 | 0.076 | 2.4 | 246-419 |
| 25 | 0.117 | 0.010 | 11.7 | 174-246 |
| 26 | 0.485 | 0.004 | 111.6 | ND |
| 27 | 0.756 | 0.254 | 3.0 | 247-419 |
| 28 | 0.682 | 0.126 | 5.4 | 374-382 |
| 29 | 0.595 | 0.003 | 198.3 | ND |
| 30 | 0.719 | 0.437 | 1.6 | ND |
| 31 | 0.764 | 0.472 | 1.6 | 1-180 |
| 32 | 0.741 | 0.413 | 1.8 | ND |
| 33 | 0.773 | 0.402 | 1.9 | ND |
| 34 | 0.561 | 0.346 | 1.6 | ND |
| 35 | 0.561 | 0.405 | 1.4 | ND |
| 36 | 0.434 | 0.003 | 144.7 | ND |
| 37 | 0.782 | 0.069 | 11.4 | ND |
| 38 | 0.630 | 0.130 | 4.8 | ND |
| 39 | 0.594 | 0.087 | 6.8 | ND |
| 40 | 0.620 | 0.098 | 6.3 | ND |

ND, not determined.
